# DEATH OF A CHILD AND MORTALITY RISK OVER THE LIFE COURSE: RACIAL DISADVANTAGE IN THE U.S.

**DOI:** 10.1093/geroni/igz038.1630

**Published:** 2019-11-08

**Authors:** Rachel Donnelly, Debra Umberson, Robert Hummer, Michael Garcia

**Affiliations:** 1 Vanderbilt University, Nashville, Tennessee, United States; 2 University of Texas, Austin, Texas, United States; 3 University of North Carolina, Chapel Hill, North Carolina, United States; 4 Population Research Center, Austin, Texas, United States

## Abstract

Numerous studies show that bereavement, including bereavement following the death of a minor child, increases mortality risk in white populations. The death of a child prior to midlife has received much less attention. Moreover, recent research shows that black Americans are substantially more likely to lose a child compared to white Americans, but this racial disadvantage is largely unexplored. Losing a child is a traumatic event that may activate biopsychosocial and behavioral risk factors that add to mortality risk. We analyze longitudinal data from the Health and Retirement Study (1992-2014) to assess the association of child loss prior to midlife with mortality risk in mid to later life, and the possible biopsychosocial and behavioral covariates linking child death to mortality. The analytic sample includes 20,489 non-Hispanic white respondents and 5,328 non-Hispanic black respondents who have ever given birth to or fathered at least one child. Findings suggest that that the death of a child prior to midlife is associated with increased mortality risk, net of sociodemographic controls. Psychological (e.g., depressive symptoms), behavioral (e.g., alcohol use, smoking), and social (e.g., income, marital status) factors explain this heightened mortality risk. Although the heightened mortality risk for child loss is similar for black and white parents, black parents experience a greater disadvantage as they are almost twice as likely as white parents to lose a child prior to midlife. Child loss and the resulting health risks disproportionately burden black families, functioning as a unique source of disadvantage for black Americans.

